# Understanding How Multisectoral Programs and Policies Across Systems Can Be Leveraged to Improve Maternal, Infant, and Child Nutrition in Guinea

**DOI:** 10.1002/hpm.70105

**Published:** 2026-07-26

**Authors:** Teresa R. Schwendler, Ramatou Hermine Sankhon, Leif Jensen, Mohamed L. Fofana, Mamady Daffé, Facely Camara, Ramakwende Zoma, Ibrahima Balde, Stephen R. Kodish

**Affiliations:** ^1^ Department of Nutritional Sciences The Pennsylvania State University University Park Pennsylvania USA; ^2^ Department of Agricultural Economics, Sociology, and Education The Pennsylvania State University University Park Pennsylvania USA; ^3^ Helen Keller Intl Conakry Guinea; ^4^ Ministère de la Santé et de l'Hygiène Publique Conakry Guinea; ^5^ Centre de Recherche et de Formation en Infectiologie de Guinée Conakry Guinea; ^6^ Department of Biobehavioral Health The Pennsylvania State University State College Pennsylvania USA

**Keywords:** infant and young child, nutrition, nutrition policy, pregnancy

## Abstract

**Objective:**

To understand how current programmes and policies can be leveraged to improve maternal, infant, and young child nutrition outcomes (MIYCN) in Guinea.

**Data Collection Methods:**

*Study design.* This study is part of a larger multiphase mixed methods study design. *Theoretical framework.* A systems framework developed by the United Nations Children's Fund (UNICEF) and an implementation science framework were used to guide this research. *Phase 1.* An in‐depth literature review of current programme and policy documents (2019–2022) targeting MIYCN through the water, sanitation, and hygiene (WASH), health, food, and social protection systems in Guinea was conducted. *Phase 2*. Semi‐structured interviews with stakeholders (*n* = 20) from each system were completed to explore stakeholders' knowledge related to pertinent programs and policies. *Phase 3.* Validation of findings was conducted using member checking.

**Data Analysis Methods:**

Grey literature documents and interview findings were analysed using content analysis guided by the systems framework and implementation science framework, respectively.

**Results:**

Policy documents across systems were all multisectoral and more than half (27/32) of evidence‐based programme modalities across systems were included in policies. Most (28/32) evidence‐based programme modalities were also being implemented in Guinea. Stakeholder interview findings revealed that programme implementation was the most salient barrier to operationalising evidence‐based programme modalities.

**Conclusion:**

Findings from our study indicate that policy and programme frameworks are strong but improved financial planning and vertical and horizontal programme planning may create a stronger enabling environment to support MIYCN in Guinea.

## Highlights

- Policy documents across systems in Guinea were all multisectoral and more than half (27/32) of evidence‐based programme modalities across systems were included in policies
- Most (28/32) evidence‐based programme modalities across systems were being implemented
- Stakeholder interview findings revealed that financing and programme planning were two of the most salient factors shaping programme implementation across systems

## 1 Introduction

Nutrition during the first 1000 days of life (conception to the child's second birthday) supports healthy growth, neurological development, immunity, and long‐term health outcomes [1]. Suboptimal macro and micronutrient intake in the short term contributes to poor linear growth, suboptimal neurological development, and altered immunity which can result in a higher risk for morbidity and mortality [2, 3]. Poor nutrition also impairs attention and working memory, which in turn result in poorer grade attainment, literacy, and incomes later in life [4, 5, 6, 7]. Infants and young children (IYC) face the greatest risk for growth faltering within the first 1000 days of life, and studies show that interventions are less likely to have as substantial of an impact on growth outcomes after this period [8].

For this reason, the United Nations Children's Fund (UNICEF) systems framework emphasises that to sustainably improve maternal, infant, and young child nutrition outcomes (MIYCN) during this critical period of growth and development, and in some cases up to the child's fifth birthday, governments must address the various determinants of nutrition across the health, food, water, sanitation, and hygiene (WASH), and social protection system [9]. Multi‐sectoral approaches, or the inclusion of programme modalities (i.e., interventions) from more than one system, have been found to have a more substantial impact on MIYCN than isolated interventions alone [10, 11, 12, 13, 14, 15].

Case studies from low‐and middle‐income countries demonstrate that key recommendations for improving the enabling environment to support multisectoral approaches include: (1) building political commitment to address undernutrition and (2) translating political commitment into programming [11]. To translate evidence into action, a clear understanding of current recommendations for multisectoral programs and a description of the current policy and programme environment within each country is needed.

Recent reviews highlight evidence‐based programme modalities that show promise for improving MIYCN across systems. The 2021 Lancet series presents strong evidence for twelve health system programme modalities that show promise for improving MIYCN, such as family planning for pregnant women and vitamin A supplementation for children up to 5 years of age [15]. A 2018 review described fortification, biofortification, homestead food production, livestock, and nutrition‐sensitive value chain programs, irrigation, and improved market access as food system entry points for improving MIYCN [16]. The 2021 Lancet series also highlights a moderate level of evidence to support WASH programme modalities, primarily due to mixed findings from observational and randomized control trials [15, 17, 18]. Finally, social protection programme modalities such as cash transfers and women's empowerment are recommended for improving MIYCN, though their impact varies [19, 20, 21, 22]. Evidence suggests that multisectoral programs and policies may help improve MIYCN in contexts where the prevalence of malnutrition is stagnant.

Turning from global evidence to the case of Guinea, stunting has affected 30% children under the age of 5 years for nearly a decade [23]. Despite strong recommendations to implement multisectoral programs to improve MIYCN, the policy and programme landscape in Guinea remains poorly described. To improve the current policy and programming environment, stakeholders first need to understand the existing environment. The present study aimed to understand how Guinea operationalised current evidence across systems to improve MIYCN by exploring existing policies and programs in Guinea and identifying opportunities to strengthen multisectoral action to improve MIYCN.

## 2 Methods

### 2.1 Study Setting

Although the UNICEF systems framework recognises four systems, multiple government offices are responsible for implementing evidence‐based programme modalities across systems in Guinea. Two multisectoral committees also coordinate activities across systems (Table 1).

**TABLE 1:** Government ministries responsible for policy and programme implementation in each system.

System	Government offices responsible for system indicators^a^
Health	• Ministry of health and public hygiene • National institute of public health
Food	• Ministry of agriculture and livestock • Ministry of fisheries, aquaculture, and maritime economy • Guinean alliance for food fortification • National quality control office
WASH	• Ministry of health and public hygiene • The Guinea water company • National institute of public health • Ministry of energy and hydraulics • Ministry of the environment, water, and forests
Social protection	• Ministry of health and public hygiene • Ministry for the advancement of women, children and vulnerable persons • National observatory of social protection and based gender violence
All	• National multisectoral nutrition committee • National committee for nutrition and food security

^a^ For indicators specific to each system see Table 4 below.

Although not outlined in this table, numerous non‐governmental organisations and United Nations partners also implement evidence‐based programme modalities across systems in Guinea.

### 2.2 Study design

We used a multiphase study design to understand the current policy and programming environment across health, food, WASH, and social protection systems in Guinea [24]. Each phase of the research was intentionally designed to inform the subsequent phase, with Phase 1 findings refining and validating Phase 2, and Phase 2 findings informing Phase 3 (Table 2).

**TABLE 2:** Multiphase iterative study design, methodological, and participant sampling frame.

Phase 1. Exploring current policies, strategies, and programs	Phase 2. Understanding factors influencing programming	Phase 3. Validating findings
Active political documents from each system^a^ (*n* = 14) Programs documents across systems^a^ published between 2019–2022 (*n* = 52)^b^	Semi‐structured interviews with stakeholders from each system^a^ (*n* = 20)	Member checking findings among in‐country stakeholders (*n* = 8)

^a^ Health, food, water, sanitation and hygiene, and social protection systems.

^b^ Not including webpages that were cited.

### 2.3 Data Collector Positionality and Training

The research team selected data collectors with a secondary education in health and who spoke French or English as a second language. The team conducted a 2‐week intensive qualitative research training to train data collectors before the study's commencement. The training covered a variety of topics, such as techniques for building rapport with participants, using open‐ended questions, strategies to reassure participants that there are no right or wrong answers, and how to accurately translate and transcribe recorded interviews. Their positionality was that of someone inside the local culture.

### 2.4 Data Collection Methods

#### 2.4.1 Phase 1. Exploring Current Policies, Strategies, and Programs

##### 2.4.1.1 Policy and Programme Review

We solicited programme and policy documents using a systematic multistep process. First, key informants from the Ministry of Health helped to develop a list of organisations working within each system in Guinea. Then, we reviewed pertinent organisations' websites and contacted stakeholders via email and over the phone to obtain relevant policy and programme documents. Other researchers have used similar approaches to obtain programme and policy documents, given that policy and programme documents are not always accessible online [25, 26]. We included documents if they were an operating policy or programme, were implemented between January 1, 2019 and December 31, 2022, and included evidence‐based programme modalities for improving MIYCN [9, 11, 14, 15, 16, 19]. For purposes of this research, we defined policy as a guideline, strategy, or legal document issued by a governing agency to set goals and provide a framework to guide the implementation of programs. We defined programs as planned interventions or activities with an explicit or implicit goal of improving MIYCN. Finally, we defined evidence‐based programme modalities as those that are supported by the current evidence to improve MIYCN [9, 11, 14, 15, 16, 19].

#### 2.4.2 Phase 2. Understanding Factors Influencing Programming

##### 2.4.2.1 Semi‐Structured Interviews With Stakeholders

We purposefully sampled stakeholders using a criterion‐based sampling approach, ensuring an equal distribution of respondents from each system, job‐positing site, and employer, to generate maximum variation in responses [27]. Because this study was part of a broader project on feeding practices in the food‐secure region of Guinea [28], we interviewed both regional representatives from Kindia and national‐level stakeholders. We determined the overall sample size (*n* = 20) a priori based on the number of interviews expected to yield data saturation [25, 29, 30, 31]. Interview guides were developed based on a review of programme and policy documents, and the list of evidence‐based programme modalities recommended for improving MIYCN [9, 11, 14, 15, 16, 19]. During the interview, data collectors probed stakeholders to describe the current policies and programs being supported by their organisation so they could be compared to the findings yielded from the document review. We also asked stakeholders to describe any factors influencing the implementation of programs and policies that their organisation supports and any recommendations to improve their impact moving forward. Trained data collectors conducted each recorded interview in French, which lasted 1–2 hours, and conducted either in person or via Zoom.

#### 2.4.3 Phase 3. Validating Findings

##### 2.4.3.1 Member Checking

Finally, we conducted a member checking exercise over the phone or by email with key informants (*n* = 8) from each system. We used member checking, a method used to verify findings [24] to ensure that findings generated from phases 1‐2 were reflective of the current policy and programme environment in Guinea. The research team also relied on the member checking exercise to solicit additional policy or programme documents that were missed during the initial grey literature search.

### 2.5 Data Analysis Methods

We used Dedoose Qualitative Analysis Software to facilitate content analysis of textual data, including programme and policy documents and interview transcripts [24, 32]. Prior to constructing the codebook, a trained data collector translated and transcribed all interviews from French into English. Throughout the translation and transcription process, random checks were conducted to ensure accuracy of the translation and transcription. We constructed the initial qualitative codebook deductively, or with codes developed based on existing evidence and theory. We used three frameworks to develop the codebook: (1) a systems framework developed by UNICEF [33], (2) a list of evidence‐based programme modalities within each system [11, 14, 15, 16, 19], and (3) an Implementation Science Framework for Nutrition (ISFN) (see Supporting Information S1: Appendix A–B) [34]. Each provisional code contained a code name, definition derived from the prior mentioned frameworks, and a description of when to use the code.

After developing the codebook, we carried out a multistep process to perform a deductive content analysis using predefined descriptive codes until data saturation was reached [24, 35]. First, we uploaded all interview transcripts and programme and policy documents to Dedoose [32]. Two researchers (TRS and RHS) conducted the first round of coding to refine the contents of the codebook, allowing for triangulation of perspectives and mitigation of bias [24]. After the first round of coding, the researchers met to refine the qualitative codes to ensure all relevant information was captured and to minimise bias [24]. One researcher (RHS), a medical doctor from Guinea fluent in English and French, was responsible for coding policy and programme documents while interview data were coded by another researcher (TRS), a PhD student and nutritionist fluent in English who lived and worked in Guinea throughout the study. We used a checklist matrix (i.e., rows representing programme and policy modalities and columns representing systems) to summarise which evidenced‐based programme modalities were present or absent in policy and programme documents [24]. We also reviewed the coded excerpts while stratifying by the domains of the ISFN framework to report on factors influencing programme and policy implementation across systems. Tables (see Supporting Information S1: Appendix C) were constructed to display the most salient, or most highly cited factors shaping programme and policies, across each ISFN implementation domain [24].

### 2.6 Ethical Approval and Compliance

The Comité National d'Ethique et de Recherche en Santé in Guinea approved the study and The Pennsylvania State University's Office of Research Protections deemed the study as exempt. Participants provided verbal informed consent prior to participating in semi‐structured interviews and were told that their names and affiliations would not be shared. Participants were told that their input would contribute to a study examining the ways multisectoral policies and programs could be leveraged to strengthen MIYCN in Guinea. Deidentified data was stored in a password‐protected electronic folder to ensure participant confidentiality was maintained.

## 3 Results

### 3.1 Participants Demographics

Interview participants represented the health (6/20, 30%), food (6/20, 30%), WASH (*n* = 3/20, 15%), and social protection (5/20, 25%) systems in Guinea. Approximately half of the respondents worked on coordinating or implementing policy or programming at the national level (10/20, 50%) and worked for a government entity (11/20, 55%). A subset of interviewed participants (*n* = 8) were contacted to validate findings during Phase 3.

### 3.2 National Policies

There were seven policies and seven strategies guiding programming across systems in Guinea. Social protection (13/14, 93%), health (11/14, 79%), and WASH (11/14, 79%) system evidence‐based‐programme modalities were more frequently included while food (9/14, 64%) system modalities were less frequently included in government policies. Five policies included programme modalities from all four systems, but all policies were multisectoral, or included evidence‐based programme modalities specific to more than one system (Table 3).

**TABLE 3:** Evidence‐based‐programme‐modalities included in operating policy documents in Guinea.

Policy or strategy		Policy targets^a^			
Title	Year(s)	Health	Food	WASH	Social protection
National social protection policy	2016–2021		✓	✓	✓
National family policy	2019–2021	✓	✓	✓	✓
National multisectoral nutrition policy	2019–2030	✓	✓	✓	✓
National agricultural development policy	2017–2025		✓		✓
National food and nutrition policy	2014–present	✓	✓	✓	✓
National health policy	2014–present	✓		✓	✓
National gender policy	2017–present	✓	✓	✓	✓
Scaling up best practices for infant and young child feeding	2021–2030	✓	✓	✓	
Strategic plan for reproductive, maternal, neonatal, infant‐juvenile, and adolescent health and nutrition	2020–2024	✓			✓
Strategic plan for community health	2018–2022	✓		✓	✓
Vision 2040 for an emerging and prosperous Guinea	2017–2040			✓	✓
National health development plan	2015–2024	✓	✓	✓	✓
Guinea's budgeted national family planning action plan	2019–2023	✓			✓
National multisectoral nutrition strategic plan	2019–2024	✓	✓	✓	✓

^a^ Policy objectives targeted one of the recommended evidence‐based programme modalities across systems.

### 3.3 Policy and Programme Mapping

**FIGURE 1:**
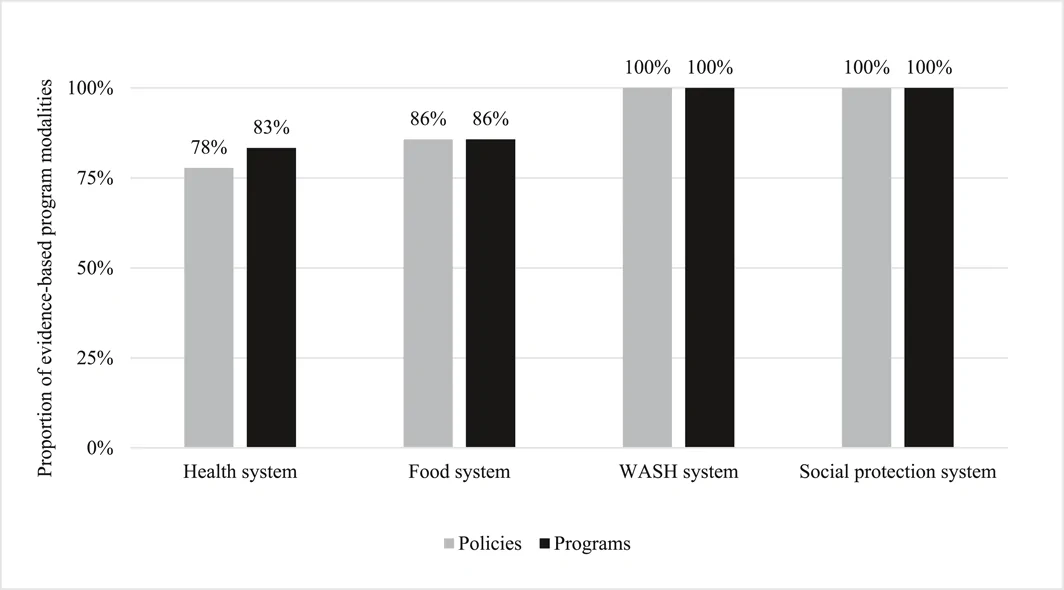
Proportion of evidence‐based programme modalities included in current policies and programmes across systems in Guinea. WASH, water, sanitation and hygiene.

More than half (27/32, 84%) of recommended MIYCN evidence‐based programme modalities across systems were included in policies. Each programme modality cited in political documents was also implemented as a programme. Most (28/32, 88%) evidence‐based programme modalities are being implemented in current programs in Guinea as of 2022 (Figure 1).

The food and social protection system programme modalities often do not directly target mothers, infants, and young children (MIYC), or do not cite MIYC as a target population of the intervention, while health and WASH system programme modalities consistently do.

### 3.4 Health System

#### 3.4.1 Health System Policies

Most programme modalities with a strong evidence base were included in health system policies (9/11, 82%) to address MIYCN [36, 37, 38, 39, 40, 41, 42, 43, 44, 45, 46]. Two programme modalities supported by strong evidence but not included in policies are the provision of Multiple Micronutrient Supplements (MMS) for pregnant women and small‐quantity lipid nutrient supplements. The provision of food baskets for food‐insecure populations was supported by policy, but pregnant women and IYC were not specifically cited as a target population of policies. Some previously recommended evidence‐based programme modalities (e.g., iron and folic acid (IFA) supplementation during pregnancy) were, included in policy documents [46] (Table 4).

**TABLE 4:** Inclusion of health system evidence‐based programme modalities in current programs and policies.

Evidence based programme modality	Evidence level based on Keats et al. [15]^a^	Policy	Programme	Programme targeted MIYCN^b^
Maternal				
Multiple micronutrients	Strong			
Calcium supplements	Moderate	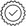 ^c^	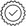 ^c^	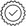 ^c^
Balanced energy protein supplementation	Moderate	✓	✓	✓
Food distribution programmes	Weak	^d^ 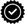	✓	✓
Family planning and birth spacing	Strong	✓	✓	✓
Infants				
Kangaroo mother care for LBW newborns	Strong	✓	✓	✓
Delayed cord clamping for preterm newborns	Strong	✓	✓	✓
Probiotics for LBW and preterm	Emerging			
Emollient use (i.e., coconut oil) for low birth weight and preterm	Emerging			
Infants and young children				
Breastfeeding promotion and counselling	Strong	✓	✓	✓
Complementary feeding education among food‐secure populations	Moderate	^d^ 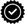	✓	✓
Complementary foods with education among food‐insecure	Strong	^d^ 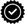	✓	✓
Vitamin a supplementation	Strong	✓	✓	✓
Therapeutic zinc supplementation for diarrhoea management	Strong	✓	✓	✓
Micronutrient powders	Moderate	✓	✓	✓
Small quantity lipid nutrient supplements	Strong		✓	✓
Ready‐to‐use supplementary food for acute malnutrition	Strong	✓	✓	✓
Across life‐stages				
Insecticide‐treated bed‐nets	Strong	✓	✓	✓

Abbreviation: MIYCN, Maternal Infant and Young Child Nutrition.

^a^ Evidence level for intervention to address maternal and child malnutrition based on the 2021 Lancet series [15].

^b^ At least one programme targeted MIYCN as the primary target population (i.e., conception to child's 5th birthday), programmes that were vague and just indicated ‘children’ or ‘women’ were the target population were not counted.

^c^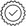 Policies and programmes that were recognized by stakeholders as being operational, but not clearly stated in policy and programme documents. Examples include calcium supplementation which is recommended in international maternity care guidelines and is provided through routine care, but not formally included in a policy or programme document, and large‐scale food fortification which is enforced by law, but we were unable to source any recent publications that highlight its implementation.

^d^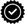 Complementary feeding education was cited in policies, but the food security status and/or age of the target population was not mentioned.

#### 3.4.2 Health System Programmes

Most (10/11, 91%) health system programme modalities with a strong evidence base are being implemented [37, 47, 48, 49, 50, 51, 52, 53, 54, 55, 56, 57, 58, 59, 60, 61, 62, 63, 64, 65]. The provision of small quantity lipid nutrient supplements is one example of a programme modality being implemented without a corresponding government policy [47, 50, 63, 66, 67]. Moderate to weak evidence‐based interventions including complementary feeding education without the provision of food, food distribution programmes, and delivery of multiple micronutrient powders for IYC, were also implemented [47, 53, 54, 63, 66]. Food distribution programmes targeted pregnant and lactating women, but often only delivered programming to those living with a specific health condition (e.g., HIV) or those classified as food insecure during the lean season [47, 63, 66]. Many programmes promoted complementary feeding without the provision of food and included educational components, but regardless of the food security status of the household did not provide concurrent food transfers [52, 53, 67, 68, 69, 70, 71, 72]. All strong evidence‐based programme modalities being implemented through the health system target MIYC, except family planning interventions, which targeted women of reproductive age [50].

### 3.5 Food System

#### 3.5.1 Food System Policies

Food fortification, the only programme modality supported by a strong evidence base, was included in policy documents and was governed by law in Guinea [37, 38, 41, 45, 73, 74]. The fortification of salt with iodine, flour with iron, zinc, and B vitamins, and oils with vitamin A is currently mandatory [37, 38, 41, 45, 73, 74]. Only one programme modality, biofortification, was not included in government policy documents (Table 5).

**TABLE 5:** Inclusion of food system evidence‐based programme modalities in current programmes and policies.

Evidence based programme modality	Evidence level based on Keats et al. [15]^a^	Policy	Programme	Programme targeted MIYCN^b^
Large‐scale food fortification	Strong	✓	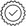 ^c^	
Biofortification	N/A^d^			
Homestead food production	N/A^d^	✓	✓	
Nutrition‐sensitive value chains	N/A^d^	✓	✓	
Livestock programming	N/A^d^	✓	✓	
Irrigation	N/A^d^	✓	✓	
Market access (e.g., *roads and market installation*)	N/A^d^	✓	✓	

Abbreviations: MIYCN, Maternal Infant and Young Child Nutrition; N/A, Not applicable.

^a^ Evidence level for intervention to address maternal and child malnutrition based on the 2021 Lancet series [15].

^b^ At least one programme targeted MIYCN as the primary target population (i.e., conception to child's 5th birthday), programmes that were vague and just indicated ‘children’ or ‘women’ were the target population were not counted.

^c^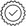 Policies and programmes that were recognized by stakeholders as being operational, but not clearly stated in policy and programme documents. Examples include calcium supplementation which is recommended in international maternity care guidelines and is provided through routine care, but not formally included in a policy or programme document and large‐scale food fortification which is enforced by law, but we were unable to source any recent publications that highlight its implementation.

^d^ N/A, Evidence on intervention modality not provided in 2021 Lancet series [15].

#### 3.5.2 Food System Programmes

Food fortification programming, the only strong evidenced‐based food system programme modality, is being implemented under the law for salt, oil, and flour in Guinea. During interviews, however, stakeholders reported that the reach of food fortification programming is variable. Other food system‐related objectives from policy documents were mirrored in programme documents except biofortification which was not cited in policy or programme documents [47, 52, 63, 66, 67, 69, 70, 71, 75, 76, 77, 78, 79, 80, 81, 82].

Many food system programmes targeted multiple food system‐specific programme modalities simultaneously, while others focused on both food and social protection programme modalities, including women's empowerment, cash transfers, or loans to farming households [47, 52, 63, 67, 69, 71, 75, 79, 83, 84, 85]. Food system programme modalities included education and resource transfers such as gardening kits, seeds, agricultural tools, fertiliser, ruminants, related infrastructure, or the connection between growers and consumers [47, 52, 63, 66, 67, 69, 70, 75, 77, 79, 83, 84, 85]. Other programmes sought to improve infrastructure related to food systems such as roads, access to water for agriculture, or the construction of new market stalls [69, 75, 84]. None of the food system programmes included in our review specifically targeted MIYCN, with the exception of nutrition education components. Instead, target populations included vulnerable households, farmers, and women.

### 3.6 WASH System

#### 3.6.1 WASH Policies

WASH programme modalities such as water access, water quality, and hygiene were all supported by government policies [37, 38, 39, 40, 41, 43, 45, 46, 81, 86, 87]. However, to our knowledge, no policy specific to water quality, sanitation, or hygiene currently exists in Guinea [88] (Table 6).

**TABLE 6:** Inclusion of WASH system evidence‐based programme modalities in current programmes and policies.

Evidence based programme modality	Evidence level based on Keats et al. [15]^a^	Policy	Programme	Programme targeted MIYCN^b^
Water access/supply	Moderate	✓	✓	✓
Water quality		✓	✓	✓
Sanitation		✓	✓	✓
Hygiene		✓	✓	✓

Abbreviations: MIYCN, Maternal Infant and Young Child Nutrition; WASH, Water, sanitation, and hygiene.

^a^ Evidence level for intervention to address maternal and child malnutrition based on the 2021 Lancet series [15].

^b^ At least one programme targeted MIYCN as the primary target population (i.e., conception to child's 5th birthday), programs that were vague and just indicated ‘children’ or ‘women’ were the target population were not counted.

#### 3.6.2 WASH Programmes

All WASH programme modalities reflected in policy documents are being implemented through programmes [52, 53, 61, 65, 68, 76, 80, 82, 83, 84, 89, 90, 91]. WASH programmes often provided broad WASH education [51, 53, 54, 82, 89] while sanitation programmes sought to end open defecation, increase solid waste management networks, and improve environmental sanitation through mass media‐based education, and recycling systems [64, 65, 68, 76, 80, 90, 91]. Environmental sanitation at the community level was described by stakeholders as being carried out by dedicated community members in rural areas, while more structured programs were noted in urban city centres.

Water access programmes sought to improve safe drinking water through establishing taps, while water quality programming provided education on the consumption of clean water and sometimes provided chorine or hygiene kits to promote water purification [51, 65, 77, 80, 89]. One programme also worked directly with the Société des Eaux de Guinée to improve water access operations in urban areas. Hygiene‐specific programme modalities primarily sought to improve hand hygiene by setting up hand washing stations in market areas, distributing soap, conducting education sessions at the community level, or education provided through radio broadcasts [52, 68, 71, 72, 83, 84]. WASH programme modalities that targeted MIYCN provided education on the importance of WASH during the complementary feeding period, provided soap, clean water canisters, or water purification kits [47, 53].

### 3.7 Social Protection System

#### 3.7.1 Social Protection Policies

All social protection programme modalities (3/3, 100%) were reflected in government strategies or policies [36, 37, 38, 39, 40, 41, 42, 43, 44, 45, 46, 73, 86] (Table 7).

**TABLE 7:** Inclusion of social protection system evidence‐based programme modalities in current programmes and policies.

Evidence based programme modality	Evidence level based on Keats et al. [15]^a^	Policy	Programme	Programme targeted MIYCN^b^
Women's empowerment (i.e., agency (e.g., decision‐making opportunities, power), resources (e.g., time, money), achievement (e.g., skills, education achievement, income earning opportunities)) [92]	N/A^c^	✓	✓	✓
Cash transfers (e.g., direct, or indirect cash transfers)	N/A^c^	✓	✓	✓
Health service vouchers (e.g., health vouchers for indigents, free maternal and child health services)	N/A^c^	✓	✓	✓

Abbreviations: MIYCN, Maternal Infant and Young Child Nutrition; N/A, Not applicable.

^a^ Evidence level for intervention to address maternal and child malnutrition based on the 2021 Lancet series [15].

^b^ At least one programme targeted MIYCN as the primary target population (i.e., conception to child's 5th birthday), programmes that were vague and just indicated ‘children’ or ‘women’ were the target population were not counted.

^c^ N/A, Evidence on intervention modality not provided in 2021 Lancet series [15].

#### 3.7.2 Social Protection Programmes

All social protection programme modalities (3/3, 100%) reflected in political documents were carried out through programmes [52, 63, 66, 69, 71, 75, 79, 82, 84, 93, 94, 95]. Women's empowerment programme modalities sought to promote opportunities for income earning opportunities, created village savings groups, credit schemes, conducted skills training (e.g., business skills, informatics, sewing, hairdressing, leadership coaching, farming techniques, and finance), and set targets for a certain proportion of their target population to be female (e.g., 50% of beneficiaries) [52, 63, 66, 69, 71, 75, 79, 82, 84, 93, 94, 95]. Health service vouchers were provided through the National Indigence Fund and other non‐government programmes to supply vulnerable populations with access to free healthcare. Routine maternal and child health (MCH) services were also provided free of cost to all families in Guinea [51]. Women's empowerment and cash transfer programmes often targeted women and farmers and not MIYCN specifically, but free routine MCH services targeted MIYCN.

### 3.8 Factors Influencing Programming and Recommendations for Improving Programmes

Various factors were described by stakeholders to shape programme implementation. However, the factors outlined below were described by stakeholders to be the most salient (i.e., important) factors influencing programmes (See Supporting Information S1: Appendix C for a complete list of factors).

#### 3.8.1 Enabling Environment

During interviews, stakeholders described policy development and funding as the most salient factors influencing programmes.

##### 3.8.1.1 Policy Development

Stakeholders perceived that some policies such as the National Multisectoral Nutrition Strategic Plan were *‘well developed’*, while others such as the National Social Protection Policy were not. Policy gaps that stakeholders recommended filling include developing: (1) a policy to guide national social protection programming, (2) regulations for water quality, including for the private sector, (3) a policy to cut widespread food insecurity during the lean season, and (4) an improved social protection mechanism for farmers. This WASH stakeholder called for more stringent water quality guidelines in Guinea to better support MIYCN.

We are currently witnessing a proliferation of factories manufacturing water in bags. Does the state have effective control of these manufacturing units to verify the quality … before putting them on the market?…This is also a factor that can have an impact on child malnutrition.

‐ WASH system stakeholder interview

##### 3.8.1.2 Funding

Regardless of whether a policy exists, funding constraints limit the application of policies across systems. This stakeholder from the health system described how the National Multisectoral Nutrition Strategic Plan (2018–2024), which includes political commitments across all four systems to improve MIYCN, has not received funding since its development in 2018.

The [National Multisectoral Nutrition Strategic] plan is going to expire [in 2024] and it is not being implemented, which is a serious problem…All this is because the country has no potential donors in the field of nutrition. Nutrition is underfunded in Guinea. It is not anyone's fault. Since the plan was developed, there has been no funding until today….

‐ Health system stakeholder interview

Given that financial constraints limit policies from being operationalised, stakeholders recommended seeking out funding as well as specifically defining programme priorities and key target populations.

#### 3.8.2 Implementing Organization

Interviewees indicated that the most salient factors shaping programmes were stakeholder technical knowledge and human resources.

##### 3.8.2.1 Stakeholder Knowledge

Stakeholders reported that at the national level, lack of cross‐system knowledge, such as the delineation between food security and nutrition, was a barrier to horizontal (i.e., across systems) stakeholder collaboration. At the decentralised or local level, recruitment of staff with inappropriate educational backgrounds coupled with a lack of funding to host training, makes it challenging for staff to do their job. This stakeholder from the social protection system described how recruitment of qualified staff can be challenging.

This recruitment is not done according to the profile [required for the job] and the state is only concerned about how to give jobs to young graduates. So, you can find a person who studies economy or medicine within the Department of Social Action, but the choice is not compatible with the department.

‐ Social protection system stakeholder interview

Capacity building across systems, especially at the decentralised level, was recommended to ensure staff have adequate knowledge to conduct their job.

##### 3.8.2.2 Human Resources

Suboptimal human resources due to funding constraints limit the reach of programmes and sometimes result in a few skilled employees being tasked with more than they can manage, such as both child vaccination and nutrition programming portfolios. This stakeholder from the health system described how suboptimal human resources and funding are both barriers to health system programming.

… maybe, when we have the personnel [to implement health system programming], we don’t have enough personnel here, there is a lack of human resources ….

‐ Health system stakeholder interview

In addition, increasing the number of trained staff was recommended, especially in the social protection and health system, to prevent staff from becoming overwhelmed and discouraged.

#### 3.8.3 Implementing Process

Across all domains, the most salient factors shaping programming were planning and scale‐up.

##### 3.8.3.1 Planning

Suboptimal planning was described to result in poor programme implementation, suboptimal use of programme funds, duplication of activities, and lack of integration of programme activities both within systems (e.g., vaccination and severe acute malnutrition screening) and across systems (e.g., social protection and health system services). Formative research to identify appropriate platforms, communication channels, and technical meetings at all levels (i.e., local, regional, national) were recommended by stakeholders to ensure programs are not duplicated and best practices are utilised. This stakeholder from the health system described how more contextually specific research is needed to understand how to translate evidence‐based programme modalities into programming.

We know the Lancet has been clear since 2007, it's over… we know how these interventions can influence the nutritional status of the child, we know. The problem is how? How do you implement it? How do we deliver it?

‐ Health system stakeholder interview

##### 3.8.3.2 Scale‐Up

Weak supply chains due to delays in ordering, funding‐related constraints, and suboptimal road conditions were described to result in delayed programme implementation and suboptimal uptake of programme activities. To improve the scale‐up of effective programme modalities, improved monitoring and evaluation systems were recommended. Strong monitoring and evaluation platforms allow for redirecting of excess stock from one programme site to another and identification of programme sites that require more support. National health and water data systems such as District Health Information Software‐2 and Notre Eau Maitrisee et Accessible may improve the scale‐up scale of programming once they are available in real‐time. However, similar monitoring platforms were recommended by stakeholders to improve programming across other programmes and systems.

#### 3.8.4 Individuals, Households, and Communities

At the individual, household, and community levels, lack of resources described as the most salient factor shaping programmes.

##### 3.8.4.1 Resources

Poor roads, especially during the rainy season, was described as limiting food‐related trade between municipalities and access to healthcare. Lack of access to basic services such as toilets, clean water, and healthcare services were described to result in community members adopting behaviours not being promoted through routine programming such as open defecation, relying on unfiltered rainwater for drinking, and use of traditional medicine. At the individual, household, and community levels, stakeholders recommended using a participatory approach to develop and implement programming. This stakeholder from the food system described how using participatory approaches to programme planning may result in better programme outcomes.

…from the outset, we must emphasise the need for the beneficiaries to be involved in the project…so that they know what we are doing … it is often said that the field [beneficiaries that] commands [the fate of the programme].

‐ Food system stakeholder interview

Stakeholders explained that by involving local non‐government organisations, government offices, and community development committees, programmes can better meet community needs while improving community ownership of programmes.

## 4 Discussion

This study provides the first comprehensive mapping of multisectoral MIYCN policies and programmes in Guinea. Through an assessment of existing policies and programmes, this study identified critical gaps and aims to provide a foundation for understanding how current efforts can support improvements in MIYCN in Guinea.

### 4.1 Implications for Policies and Programmes

First, findings revealed that all policies were multisectoral. However, the Hunger and Nutrition Commitment Index (HANCI), an indicator that assesses public spending, policies, laws, and programme implementation related to hunger and nutrition using 22 indicators, ranks Guinea in the bottom 25% across sub‐Saharan Africa. The HANCI also ranks Guinea well below neighbouring countries, such as Burkina Faso, which has one of the highest levels of commitment in sub‐Saharan Africa [96]. Political frameworks across systems in Guinea show promise given the growing consensus that a multisectoral approach is needed to improve MIYCN, but certain HANCI indicators act as facilitators and barriers to nutrition‐related political commitments [9, 10, 11, 97, 98, 99]. For example, the establishment of a multisectoral coordination committee and the inclusion of time‐bound health targets in existing policies boost Guinea's rank [96]. However, public spending on health (6%) and agriculture (2.3%) remains well below the recommended allotments of 15% and 10%, respectively [96, 100]. WASH objectives have been incorporated into policies across systems based on the present review, but national indicators remain poor, with only 22.7% and 79.9% of the Guinean population having access to improved water and sanitation, another contributor to poor HANCI rank [96]. Finally, gender equity‐related objectives are outlined in policies, but not implemented in practice [96]. Although our study found that policies are multisectoral, public spending budgets and poor programme implementation are two factors that may be limiting the impact of multisectoral policies on MIYCN.

Interview findings revealed that financing was a salient factor across programme implementation domains in Guinea. The Scaling Up Nutrition (SUN) movement offers several key recommendations for financial planning that can help address persistent financial barriers in Guinea. First, SUN recommends creating a clear list of programmes, such as those included in Guinea's National Multisectoral Nutrition Strategic Plan [101, 102]. Next, the cost of implementing each programme should be assessed to understand funding gaps and to create realistic programming goals [102, 103, 104, 105, 106, 107]. The Ministry of Health in Guinea has already costed and prioritised health system programmes, but realistic estimates are needed for other systems [100]. In some cases, available budgets may not be sufficient to roll out every programme nationally, and in these cases, identifying key target programmes and populations are needed [102, 108]. Routine and transparent tracking of nutrition‐related financial investments is also recommended once budgets are established [102, 109]. A great example of routine and transparent tracking is the Ministry of Economy and Finance in Peru, where ministry budgets are available online to enable users to aggregate funds across different levels of government to facilitate programme planning [110]. Finally, SUN recommends advocacy to secure funding, potentially through public‐private partnerships, to address identified gaps in programme budgets [101, 107, 109, 111, 112]. Since 2018, the National Multisectoral Nutrition Strategic Plan has received little funding; leveraging SUN guidance may be one way to improve the enabling environment to support MIYCN in Guinea.

Programme planning was also described as a salient factor shaping programme implementation by stakeholders during interviews. Best practices from other SUN member countries could be used to help inform programme planning in Guinea [101]. One best practice to strengthen programme planning is creating multisectoral programme implementation roadmaps at the national, subnational, and community levels [113, 114]. While national roadmaps exist in Guinea, adapting these plans to the subnational level is crucial for ensuring they are practical at decentralised levels [115]. Countries that have been successful in implementing multisectoral nutrition plans at the subnational level have introduced very few new programmes and instead focused on establishing programme coherence mechanisms [116, 117]. Examples of programme coherence mechanisms include identifying key target beneficiaries, such as all children diagnosed with stunting or all households with children under 2 years of age, to receive a multisectoral programme package [116, 117]. The Integrating Nutrition in Value Chains (INVC) project conducted in Malawi, which reduced the prevalence of stunting among children under 5 years by 11.6% between 2012 and 2016, is a great example [118]. INVC used care groups to provided health screening services and nutrition education (e.g., food processing, gardening), and agricultural groups to increase crop diversification, storage, processing, and marketing of nutritious foods. Given that different organisations are working in each geographic area in Guinea, developing carefully crafted roadmaps to guide multisectoral programme implementation at the subnational level may be one way to improve MIYCN [119].

Our review of programmes revealed that MMS, one key programme modality with a strong level of evidence, is not included in current policies or programs in Guinea. Given the poor rates of antenatal care (ANC) coverage (35% of women attended 4+ ANC visits) [120] and high rates of anaemia (48% of pregnant women) [121] in Guinea, the introduction of MMS has the potential to have a great impact on MIYCN in the next five to 10 years [15]. In 2020, the WHO ‘*recommended* [MMS including iron and folic acid] *in the context of rigorous research*’ as part of ANC [122]. In 2021, WHO added MMS to the list of essential medicines [123], which is a key step in the transition from IFA to MMS. Although few countries worldwide have implemented MMS at scale, there are eight key recommendations for country‐level introduction of MMS [124]. Programmatic recommendations include: (1) creating an enabling environment to support MMS, (2) analysing key determinants of anaemia, 3) introducing MMS as part of comprehensive ANC package, (4) ensuring adequate supply, (5) strengthening health workers capacity, (6) developing and integrating tailored behavioural change communication, (7) ensuring anaemic women are being identified and treated, and (8) integrating MMS indicators into national monitoring systems [124]. Extensive formative research will be needed to inform the introduction of MMS in Guinea, including identifying strategies to overcome frequently experienced hurdles such as supply chain bottlenecks and stockouts [122]. Introduction to MMS is a huge undertaking for any health system and thus, may be something that the Ministry of Health works towards implementing over the next five to 10 years.

### 4.2 Strengths and Limitations

This study had several strengths and limitations. A strength of this study was inclusion of both grey literature documents and qualitative interview data, which allowed for triangulation of findings across data sources [125]. Second, this study used member checking, whereby stakeholders from each system reviewed findings to ensure that the researcher's interpretations aligned with what was reported [27]. One limitation of our study was that barriers and facilitators to programme implementation were generated from 20 regional and national stakeholders [92]. Regional‐level stakeholders were sampled from the Kindia region as part of a larger multiphase mixed methods study, and thus, the regional‐level perspectives included in this research may not be generalisable to other regions in Guinea. Secondly, the scale and impact of current programs were not assessed, primarily because current programme documents in Guinea did not provide sufficient information. Future documents that summarise programs in Guinea should strive to describe their scale and impact.

## 5 Conclusion

Findings from this study revealed a few key recommendations to improve MIYCN in Guinea. First, conducting a costing exercise of evidence‐based programs across systems may help prioritise programs and populations that will benefit most from a multisectoral programme package. Second, designing multisectoral programme road maps at both the national and subnational levels may help improve the implementation of evidence‐based programs and MIYCN. Finally, in the next five to 10 years, rigorous formative research should be conducted to inform the introduction and scale‐up of MMS.

## Funding

Funding was received from the Fulbright U.S. Student Programme and The Pennsylvania State University. The Pennsylvania State University granted funds to support this research through multiple endowments including Beard Programme Endowment, Nutritional Sciences Departmental Endowment Award, Mary Frances Picciano Endowment Award, College of Health and Human Development Endowment Grant, and the Ann Atherton Hertzler Endowment.

## Conflicts of Interest

The authors declare no conflicts of interest.

## Supporting information


Supporting Information S1

## Data Availability

The data that support the findings of this study are available from the corresponding author upon reasonable request.

## References

[1] C. G. Victora , L. Adair , C. Fall , et al., “Maternal and Child Undernutrition: Consequences for Adult Health and Human Capital,” Lancet 371, no. 9609 (2008): 340–357, 10.1016/s0140-6736(07)61692-4. 18206223 PMC2258311

[2] I. Olofin , C. M. McDonald , M. Ezzati , et al., “Associations of Suboptimal Growth With All‐Cause and Cause‐Specific Mortality in Children Under Five Years: A Pooled Analysis of Ten Prospective Studies,” PLoS One 8, no. 5 (2013): e64636, 10.1371/journal.pone.0064636. 23734210 PMC3667136

[3] M. J. Rytter , L. Kolte , A. Briend , H. Friis , and V. B. Christensen , “The Immune System in Children With Malnutrition—A Systematic Review,” PLoS One 9, no. 8 (2014): e105017, 10.1371/journal.pone.0105017. 25153531 PMC4143239

[4] B. R. Kar , S. L. Rao , and B. A. Chandramouli , “Cognitive Development in Children With Chronic Protein Energy Malnutrition,” Behavioral and Brain Functions 4, no. 1 (2008): 31, 10.1186/1744-9081-4-31. 18652660 PMC2519065

[5] D. Pizzol , F. Tudor , V. Racalbuto , A. Bertoldo , N. Veronese , and L. Smith , “Systematic Review and Meta‐Analysis Found That Malnutrition Was Associated With Poor Cognitive Development,” Acta Paediatrica 110, no. 10 (2021): 2704–2710, 10.1111/apa.15964. 34077582

[6] A. J. Prendergast and J. H. Humphrey , “The Stunting Syndrome in Developing Countries,” Paediatrics and International Child Health 34, no. 4 (2014): 250–265, 10.1179/2046905514y.0000000158. 25310000 PMC4232245

[7] E. A. Hanushek and L. Woessmann , “The Role of Cognitive Skills in Economic Development,” Journal of Economic Literature 46, no. 3 (2008): 607–668, 10.1257/jel.46.3.607.

[8] C. G. Victora , M. de Onis , P. C. Hallal , M. Blössner , and R. Shrimpton , “Worldwide Timing of Growth Faltering: Revisiting Implications for Interventions,” Pediatrics 125, no. 3 (2010): e473–e480, 10.1542/peds.2009-1519. 20156903

[9] United Nations Children’s Fund (UNICEF) , Improving Young Children’s Diets During the Complementary Feeding Period (UNICEF, 2020).

[10] N. J. Haselow , A. Stormer , and A. Pries , “Evidence‐Based Evolution of an Integrated Nutrition‐Focused Agriculture Approach to Address the Underlying Determinants of Stunting,” supplement, Maternal and Child Nutrition 12, no. S1 (2016): 155–168, 10.1111/mcn.12260. 27187913 PMC5084820

[11] S. Gillespie , J. Hoddinott , N. Nisbett , S. Arifeen , and M. van den Bold , “Evidence to Action: Highlights From Transform Nutrition Research (2012‐2017),” Food and Nutrition Bulletin 39, no. 3 (2018): 335–360, 10.1177/0379572118788155. 30079765

[12] M. T. Ruel , A. R. Quisumbing , and M. Balagamwala , Nutrition‐Sensitive Agriculture: What Have We Learned and Where Do We Go From Here? (2017).

[13] J. Manley , Y. Balarajan , S. Malm , et al., “Cash Transfers and Child Nutritional Outcomes: A Systematic Review and Meta‐Analysis,” BMJ Global Health 5, no. 12 (2020): e003621, 10.1136/bmjgh-2020-003621. PMC7751217 33355262

[14] J. Manley , H. Alderman , and U. Gentilini , “More Evidence on Cash Transfers and Child Nutritional Outcomes: A Systematic Review and Meta‐Analysis,” BMJ Global Health 7, no. 4 (2022): e008233, 10.1136/bmjgh-2021-008233. PMC8977747 35365481

[15] E. C. Keats , J. K. Das , R. A. Salam , et al., “Effective Interventions to Address Maternal and Child Malnutrition: An Update of the Evidence,” Lancet Child & Adolescent Health 5, no. 5 (2021): 367–384, 10.1016/s2352-4642(20)30274-1. 33691083

[16] M. T. Ruel , A. R. Quisumbing , and M. Balagamwala , “Nutrition‐Sensitive Agriculture: What Have We Learned So Far?,” Global Food Security 17 (2018): 128–153, 10.1016/j.gfs.2018.01.002.

[17] A. D. Dangour , L. Watson , O. Cumming , et al., “Interventions to Improve Water Quality and Supply, Sanitation and Hygiene Practices, and Their Effects on the Nutritional Status of Children,” Cochrane Database of Systematic Reviews 2013, no. 8 (2013), 10.1002/14651858.cd009382.pub2. PMC11608819 23904195

[18] N. Akseer , H. Tasic , M. Nnachebe Onah , et al., “Economic Costs of Childhood Stunting to the Private Sector in Low‐ and Middle‐Income Countries,” eClinicalMedicine 45 (2022): 101320, 10.1016/j.eclinm.2022.101320. 35308896 PMC8927824

[19] R. A. Heidkamp , E. Piwoz , S. Gillespie , et al., “Mobilising Evidence, Data, and Resources to Achieve Global Maternal and Child Undernutrition Targets and the Sustainable Development Goals: An Agenda for Action,” Lancet 397, no. 10282 (2021): 1400–1418, 10.1016/s0140-6736(21)00568-7. 33691095

[20] L. C. Fernald , P. J. Gertler , and L. M. Neufeld , “Role of Cash in Conditional Cash Transfer Programmes for Child Health, Growth, and Development: An Analysis of Mexico's Oportunidades,” Lancet 371, no. 9615 (2008): 828–837, 10.1016/s0140-6736(08)60382-7. 18328930 PMC2779574

[21] A. Ahmed , J. Hoddinott , and S. Roy , Food Transfers, Cash Transfers, Behavior Change Communication and Child Nutrition (International Food Policy Research Institute (IFPRI), 2019).

[22] B. Fenn , T. Colbourn , C. Dolan , S. Pietzsch , M. Sangrasi , and J. Shoham , “Impact Evaluation of Different Cash‐Based Intervention Modalities on Child and Maternal Nutritional Status in Sindh Province, Pakistan, at 6 Mo and at 1 Y: A Cluster Randomised Controlled Trial,” PLoS Medicine 14, no. 5 (2017): e1002305, 10.1371/journal.pmed.1002305. 28542506 PMC5441577

[23] Global Nutrition Report. (2023), https://globalnutritionreport.org/resources/nutrition‐profiles/africa/western‐africa/guinea/.

[24] M. B. Miles , A. M. Huberman , and J. Saldaña , Qualitative Data Analysis: A Methods Sourcebook, 3rd ed. (SAGE Publications, Inc., 2014).

[25] O. Ouedraogo , M. H. Doudou , K. M. Drabo , et al., “Policy Overview of the Multisectoral Nutrition Planning Process: The Progress, Challenges, and Lessons Learned From Burkina Faso,” International Journal of Health Planning and Management 35, no. 1 (2020): 120–139, 10.1002/hpm.2823. 31271224

[26] S. S. P. Godakandage , U. Senarath , H. S. Jayawickrama , et al., “Policy and Stakeholder Analysis of Infant and Young Child Feeding Programmes in Sri Lanka,” BMC Public Health 17, no. 2 (2017): 522, 10.1186/s12889-017-4342-4. 28675132 PMC5496021

[27] M. Q. Patton , Qualitative Research & Evaluation Methods: Integrating Theory and Practice, 4th ed. (SAGE Publications, 2015).

[28] T. R. Schwendler , K. L. Keller , L. Jensen , et al., “Caregiver Feeding Practices in Guinea: Implications for Infant Dietary Diversity,” Maternal and Child Nutrition 21, no. 3 (2025): e70017, 10.1111/mcn.70017. 40108478 PMC12150137

[29] G. C. Klemm , R. Kayanda , A. Kazoba , J. McCann , L. P. Nnally , and K. L. Dickin , “Translating Multisectoral Nutrition Policy into Community Practice: Participation of Nutrition Officers in Tanzania Fosters Effective Collaborative Strategies to Improve Child Nutrition,” Current Developments in Nutrition 6, no. 4 (2022): nzac030, 10.1093/cdn/nzac030. 35415387 PMC8992576

[30] S. C. Masefield , A. Msosa , F. K. Chinguwo , and J. Grugel , “Stakeholder Engagement in the Health Policy Process in a Low Income Country: A Qualitative Study of Stakeholder Perceptions of the Challenges to Effective Inclusion in Malawi,” BMC Health Services Research 21, no. 1 (2021): 984, 10.1186/s12913-021-07016-9. 34537033 PMC8449519

[31] H. Alderman , L. Elder , A. Goyal , et al., Improving Nutrition Through Multisectoral Approaches (2016).

[32] SocioCultural Research Consultants LLC , Dedoose Version 8.0.35, (2018).

[33] UNICEF , “Improving Young Children’s Diets During the Complementary Feeding Period,” in UNICEF Programming Guidance (UNICEF, 2020).

[34] A. Tumilowicz , M. T. Ruel , G. Pelto , et al., “Implementation Science in Nutrition: Concepts and Frameworks for an Emerging Field of Science and Practice,” Current Developments in Nutrition 3, no. 3 (2019): nzy080, 10.1093/cdn/nzy080. 30864563 PMC6400593

[35] M. B. Miles , A. M. Huberman , and J. Saldaña , Qualitative Data Analysis: A Methods Sourcebook (SAGE, 2014).

[36] Ministere de l'action sociale de la promotion feminine et de l'enfance Politique Familiale Nationale (2019).

[37] Ministry of Health: Republic of Guinea , National Strategic PlanMultisectoral Nutrition (PSNMN) 2019‐2024 (2018).

[38] Republique de Guinee , Politique Nationale d’Alimentation et de Nutrition (2014).

[39] 39. Ministere de la Sante et de l'hygiene Publique . Politique Nationale de Sante (2014).

[40] Politique nationale genre revisee , Ministère de l’Action Sociale, de la Promotion Féminine et de l’Enfance (2017).

[41] Ministere de la Santé , Plan de passage a l’echelle des pratiques optimales d’alimentation du nourrisson et du jeune enfant (Direction nationale de la sante familliale et de la nutrition: Division alimentation et nutrition (2021).

[42] Ministère de la Santé , “Plan Strategique SRMNIA‐N,” in Direction Nationale de la Santé Familiale et de la Nutrition, (2019).

[43] Direction Nationale de la Santé Communautaire et de la Médecine Traditionnelle , Plan strategique de sante communautaire (Ministere de la sante, 2018).

[44] Republique de guinee ministère de la santé direction nationale de la sante familiale et de la nutrition,” Plan d’Action National Budgétisé de Planification Familiale de la Guinée, (2019).

[45] Ministere de la Sante et de l'hygiene Publique , “Plan Stratégique National Multisectoriel de Nutrition (PSNMN) 2019‐2024,” (2019).

[46] Ministere de la Sante et de l'hygiene Publique , Plan National de Developpement Sanitaire 2015‐2024. Conakry (Guinea Ministry of Health, 2015).

[47] WFP , Annual Country Report 2021 Conakry, Guinea (WFP, 2021), https://www.wfp.org/publications/annual‐country‐reports‐guinea.

[48] UNFPA , “United Nations Population Fund Country Programme Document for Guinea 2017‐2022 New York, USA: Executive Board of the United Nations Development Programme, the United Nations Population Fund and the United Nations Office for Project Services,” (2017), https://www.unfpa.org/data/transparency‐portal/unfpa‐guinea.

[49] USAID , Country Development Cooperation Strategy (CDCS), (2020).

[50] J. USAID , Projet Haute qualité des services de Santé pour le Développement HSD (2022).

[51] The World Bank , Guinea Health Service and Capacity Strengthening Project (World Bank, 2018).

[52] Child Fund Annual Report (Child Fund, 2022).

[53] UNICEF , République de Guinée (2022).

[54] UNICEF , “Country Office Annual Report 2021,” (2021), https://www.unicef.org/reports/country‐regional‐divisional‐annual‐reports‐2021/Guinea.

[55] HKI , Rapport annuel des activités de Supplémentation en vitamine A (HKI, 2021).

[56] HKI , Rapport annuel sur la Supplémentation en vitamine A (HKI, 2020).

[57] Nutiriton International , “Improving Child Survival Rates With Twice‐Yearly Vitamin A Supplementation,” (2021), https://www.nutritionintl.org/project/vitamin‐a‐supplementation/.

[58] Ministere de la Sante et de l’hygiene Publique , Rapport annuel 2022 de la direction nationale de la sante familiale et dela nutrition. Conakry: Guinea2022.

[59] UNICEF , 2022 Guinea Conakry—SAM Management, ed. UNICEF (2022).

[60] RIT International , “Usaid’s StopPalu Project Achieved Significant Gains Against Guinea’s Leading Cause of Illness and Death 2017,”, https://www.rti.org/impact/usaid‐stoppalu‐project‐fights‐malaria‐guinea.

[61] USAID. StopPalu+ President’s Malaria Initiative (PMI) , Program Component (USAID, 2020).

[62] Global Fund , Fraudulent and Abusive Practices in LLIN Mass Campaign, 2023).

[63] WFP , Annual Country Report 2022 (WFP, 2022).

[64] UNICEF , Country Office Annual Report 2022 (UNICEF, 2022).

[65] UNICEF , Country Office Annual Report 2020 (UNICEF, 2020).

[66] WFP , Annual Country Report 2020 (WFP, 2020), https://www.wfp.org/publications/annual‐country‐reports‐guinea.

[67] WFP , Annual Country Report: 2019 (WFP, 2019), https://www.wfp.org/publications/annual‐country‐reports‐guinea.

[68] OPALS , “Projet D’Amélioration de la santé maternelle et infantile avec mise à disposition d’une offre de PTME du VIH de qualité dans la Préfecture de Télimélé,” (OPALS, 2021).

[69] FIDA. Projet pour l’Agriculture Familiale , “Résilience et Marché en Haute et Moyenne Guinée (AgriFARM),” in Rapport de conception finale et appendices (2018).

[70] FIDA . Projet visant l’agriculture familiale, la résilience et les marchés enHaute et Moyenne Guinée Rapport de supervision (FIDA, 2021).

[71] GRET , Créer des synergies d’acteurs pour la transition vers des systèmes alimentaires durables et favorables à la nutrition en guinée (GRET, 2023).

[72] GRET , Les organisations paysannes et la nutrition (2021).

[73] Ministère de l’Agriculture MdlEedPA, Ministère des Pêches, de l’Aquaculture et de l’Economie Maritime, Ministère de l’Environnement, des Eaux et Forêts , Politique nationale de développement agricole (2017).

[74] Republique de Guinee , Manuel des normes et procedures de prévention et contrôle des carences en micronutriments a l’usage des agents de sante et acteurs communautaires (2020).

[75] FIDA , Programme d'options stratégiques pour le pays (FIDA, 2020).

[76] AFD , “AFD and Guinea: Tackling Poverty and Supporting a Well‐Balanced Growth,” (2023), https://www.afd.fr/en/page‐region‐pays/guinea.

[77] JICA , “Development Through Sustainable Use of Abundant Resources,” (2019), https://www.jica.go.jp/english/africahiroba/activities/activities_191219_03.html.

[78] ENABEL , “Développement de l’Entrepreneuriat Agricole sur l’axe Conakry‐Kindia‐Mamou (DEA‐CKM,” (2018), https://open.enabel.be/en/GIN/2292/p/dveloppement‐de‐l‐entrepreneuriat‐agricole‐sur‐l‐axe‐conakry‐kindia‐mamou‐dea‐ckm.html.

[79] BEDEA Rapport Annuel (2019).

[80] BADEA Rapport Annuel: 2020 (BADEA, 2020).

[81] Le CILSS , 50 ans d'engagement au service des populations saheliennes et ouest‐africanines (2023).

[82] The World Bank Group , Country Partnership Framework for the Republic of Guinea: 2018‐2023 (2019).

[83] WFP , WFP Guinea Country Brief (WFP, 2022), https://www.wfp.org/countries/guinea.

[84] FIDA . Projet visant l’agriculture familiale, la résilience et les marchés en Haute et Moyenne Guinée (FIDA, 2021).

[85] WFP , Plan stratégique de pays provisoire (2019).

[86] Ministère du Plan et de la Coopération Internationale , Vision 2040 pour une Guinée émergente et prospère (2017).

[87] Ministère de l’Action Sociale de la Promotion Féminine et de l’Enfance , Document de politique nationale de protection sociale (2016), https://socialprotection.org/fr/discover/legal_policy_frameworks/document‐de‐politique‐nationale‐de‐protection‐sociale‐0.

[88] World Health Organization , Guidelines for Drinking‐Water Quality (2022).

[89] AGIL , Plan Strategique (AGIL, 2018).

[90] ENABEL , Programme de développement et d’assainissement urbain en Guinée—communes périphériques de Conakry—SANITA Villes Propres 2 (2021), https://open.enabel.be/en/GIN/2408/p/programme‐de‐dveloppement‐et‐d‐assainissement‐urbain‐en‐guine‐communes‐priphriques‐de‐conakry‐sanita‐villes‐propres‐2.html.

[91] ENABEL , Programme de Développement et d’Assainissement Urbain en Guinée (SANITA)—Villes Propres (2018), https://open.enabel.be/en/GIN/2275/p/programme‐de‐dveloppement‐et‐d‐assainissement‐urbain‐en‐guine‐sanita‐villes‐propres.html.

[92] P. Webb , S. Ghosh , R. Shrestha , et al., “Measuring Nutrition Governance:An Analysis of Commitment,” supplement, Capability, and Collaboration in Nepal 37, no. S4 (2016): S170–S182, 10.1177/0379572116674856. 27909261

[93] République de Guinée , Centre d’Autonomisation et d’Entrepreneuriat Féminin (2023), https://caf‐guinee.com/.

[94] UNFPA , UNFPA Guinea (2023), https://www.unfpa.org/data/transparency‐portal/unfpa‐guinea.

[95] ENABEL , Développement de l’Entrepreneuriat Féminin sur l’axe Conakry‐Kindia‐Mamou (DEF‐CKM, 2018), https://open.enabel.be/en/GIN/2371/p/dveloppement‐de‐l‐entrepreneuriat‐fminin‐sur‐l‐axe‐conakry‐kindia‐mamou‐def‐ckm.html.

[96] HANCI , “Key Data for Guinea 2022,”, https://archive.ids.ac.uk/hanci/africa.hancindex.org/countries/guinea/index.html.

[97] Scaling Up Nutrition , MQSUN+ Toolkit on Multi‐Sectoral Planning for Nutrition (2020), https://scalingupnutrition.org/news/mqsun‐toolkit‐multi‐sectoral‐planning‐nutrition.

[98] The World Bank , Improving Nutrition Through Multisectoral Approaches (World Bank, 2013).

[99] Z. A. Bhutta , N. Akseer , E. C. Keats , et al., “How Countries Can Reduce Child Stunting at scale: Lessons From Exemplar Countries,” supplement, American journal of clinical nutrition 112, no. S2 (2020): 894s–904s, 10.1093/ajcn/nqaa153. 32692800 PMC7487427

[100] Ministere de la Sante Direction Nationale de la Sante Familiale et de la Nutrition , Dossier d’investissement pour la reduction de la mortalite maternelle, neonatale, infanto juvenile et des adolescents (2020).

[101] Scaling Up Nutrition , “SUN Countries: Guinea,” (2023), https://scalingupnutrition.org/sun‐countries/guinea.

[102] S. Kaufmann , T. Walters , B. Koloshuk , et al., MODULE 4: Costing and Financing for Nutrition (Path, 2020).

[103] B. Jhu and M. Gates , “The Lives Saved Tool,” (2023), https://www.livessavedtool.org/.

[104] WHO , OneHealth Tool (2023), https://www.who.int/tools/onehealth.

[105] Optima Consortium for Decision Science , OptimaNutrition (2021), http://optimamodel.com/nutrition/.

[106] Europian Comission , Inspiring the Shift from Nutrition policy to Implementation: How Existing Data Can Support Nutrition decision‐making in Guatemala (Directorate‐General International Cooperation and Development, 2019).

[107] A. Pomeroy‐Stevens , A. D'Agostino , N. Adero , et al., “Prioritizing and Funding the Uganda Nutrition Action Plan,” supplement, Food and Nutrition Bulletin 37, no. S4 (2016): S124–S141, 10.1177/0379572116674554. 27909258

[108] M. H. Doudou , O. Ouedraogo , B. Ouaro , N. Bidault , K. J. F. Reinhardt , and n bulletin , “Mapping Nutrition Interventions, a Key Analytical Tool for Informing the Multisectoral Planning Process: Example From Burkina Faso,” Food and Nutrition Bulletin 39, no. 3 (2018): 449–464, 10.1177/0379572118782881. 30238803

[109] A. M. Acosta and J. Fanzo , Fighting Maternal and Child Malnutrition: Analysing the Political and Institutional Determinants of Delivering a National Multisectoral Response in Six Countries (2012).

[110] Ministry of Economy and Finance , Integrated Financial Management Information System (2023), https://apps5.mineco.gob.pe/bingos/seguimiento_pi/Navegador/default.aspx.

[111] République de Guinée , Stratégie de plaidoyer et de communication pour la nutrition en République de Guinée (2019).

[112] J. Hoddinott , S. Gillespie , and S. Yosef , “Public‐Private Partnerships and Undernutrition: Examples and Future Prospects,” World Review of Nutrition & Dietetics 115 (2016): 233–238, 10.1159/000442110. 27198661

[113] Republic of Madagascar , Madagascar Plan National D'Action pour la Nutrition (2017‐2021).

[114] E. B. P. Ogada , N. Lelijveld , N. Sessions , G. Desplats , and K. T , “Multi‐Sectoral Nutrition Programming: Exploring Impact,” 67 (May 2022), https://www.ennonline.net/fexdigest/67/multisectoralprogramming.

[115] A. Coile , J. Wun , M. T. Kothari , C. Hemminger , P. Fracassi , and D. Di Dio , “Scaling Up Nutrition Through Multisectoral Planning: An Exploratory Review of 26 National Nutrition Plans,” Maternal and Child Nutrition 17, no. 4 (2021): e13225, 10.1111/mcn.13225. 34101997 PMC8518575

[116] S. Kaufmann , T. Walters , B. Koloshuk , et al., MODULE 6: Preparing for Inception and Implementation (PATH, 2020).

[117] SUN KM Team , “Sub‐National, Multi‐Sector Nutrition Programming: Key Findings From Eight Country Case Studies,” (2020): [cited Global], https://www.ennonline.net/mspsynthesis2020.

[118] DAI , Malawi—Feed the Future Integrating Nutrition Into Value Chains Internet (DAI), https://www.dai.com/our‐work/projects/malawi‐integrating‐nutrition‐value‐chains?utm_source=chatgpt.com.

[119] Republic of Guinea , Politique Nationale Multisectorielle de Nutrition 2019‐2030 (2018).

[120] Institut National de la Statistique Ministère du Plan et du Développement Economique. (République de Guinée: Enquête Démographiqueet de Santé, 2019), http://dhsprogram.com/pubs/pdf/FR353/FR353.pdf.

[121] K. Weze , A. I. Abioye , C. Obiajunwa , and M. Omotayo , “Spatio‐Temporal Trends in Anaemia Among Pregnant Women, Adolescents and Preschool Children in Sub‐Saharan Africa,” Public Health Nutrition 24, no. 12 (2021): 3648–3661, 10.1017/s1368980020004620. 33190664 PMC10195357

[122] WHO , “WHO Antenatal Care Recommendations for a Positive Pregnancy Experience,” in Nutritional Interventions Update: Multiple Micronutrient Supplements During Pregnancy (World Health Organization, 2020): viii. 32783435

[123] WHO , World Health Organization Model List of Essential Medicines—22nd List (World Health Organization2021.

[124] Multiple Micronutrient Supplementation in Pregancy Technical Advisory Group U, Micronutrient Forum, Nutrition International, Vitamin. Angels , Interim Country‐Level Decision‐Making Guidance for Introducing Multiple Micronutrient Supplementation for Pregnant Women (2020), https://www.unicef.org/media/96971/file/Interim‐Country‐level‐Guidance‐MMS‐for‐PW‐2021.pdf#:~:text=Nutritional%20interventions%20update%3A%20Multiple%20micronutrient%20supplements%20during%20pregnancy1,research%20is%20recommended%20to%20address%20remaining%20clinical%20questions.

[125] M. Q. Patton , “Enhancing the Quality and Credibility of Qualitative Analysis,” Health Services Research 34, no. 5 Pt 2 (1999): 1189–1208. 10591279 PMC1089059

