# Supplementary material for: Understanding How Multisectoral Programs and Policies Across Systems Can Be Leveraged to Improve Maternal, Infant, and Child Nutrition in Guinea
Source: Int J Health Plann Manage. 2026 Jul 26;41(5):1071–84. doi: 10.1002/hpm.70105 (PMC13551520; doi:10.1002/hpm.70105)
Supplement: Supplementary file 1 [file HPM-41-1071-s001.docx]

**Appendix A. Qualitative codebook - Program and policies**

| **Code no.** | **Brief code name** | **Definition of evidence-based recommendation** | **When to use code**  ** Should be used to code program summary* |
| --- | --- | --- | --- |
| **1.0** | **Health system programing** |  |  |
| **1.1** | **Maternal** |  |  |
| **1.1.1** | **Multiple micronutrient** | Programs that seek to improve multiple micronutrient supplementation among pregnant women. This can also involve acceptance trials or formative research to inform interventions.^1^  **Supplement names include:**  -Multiple Micronutrient Supplements | **Intervention examples:**   - Education of health workers, community workers or caregivers on importance of multiple micronutrient supplements and the importance of compliance for a healthy pregnancy - Pilot intervention to determine acceptability of multiple micronutrients among pregnant women - Intervention at the facility level to distribute multiple micronutrients to pregnant women - Intervention to improve the supply chain of multiple micronutrients so they can be easily reached by rural and urban health posts |
| **1.1.2** | **Iron and folic acid** | Programs that seek to improve iron and folic acid supplementation among women of reproductive age and pregnant women  **Supplement names include:**   - IFA (iron and folic acid) | **Intervention examples:**   - Education of health workers, community workers or caregivers on importance of iron and folic acid to prevent neural tube defects and anemia and the importance of compliance for a healthy pregnancy - Pilot intervention to determine new distribution or educational approaches for iron and folic acid among pregnant women - Intervention at the facility level to distribute iron and folic acid to pregnant women - Intervention to improve the supply chain of iron and folic acid so they can be easily reached by rural and urban health posts |
| **1.1.3** | **Calcium supplementation** | - Programs that seek to improve calcium supplementation for women of reproductive age and pregnant woman^1^ | **Intervention examples:**   - Education of health workers, community workers or caregivers on importance of calcium to prevent pre-eclampsia or eclampsia during pregnancy - Intervention at the facility level to distribute calcium to pregnant women - Intervention to improve the supply chain for calcium supplements so they can be easily reached by rural and urban health posts |
| **1.1.4** | **Balanced energy protein supplementation** | - Protein energy supplements provided to pregnant women during pregnancy^1^   **Supplement names include:**   - Lipid nutrient supplements (LNS) - Small quantity lipid nutrient supplements (SQ-LNS) - Micronutrient enriched porridge - CSB++ or CBS+ | **Intervention examples:**   - Education of health workers, community workers or caregivers on importance of LNS/SQ-LNS etc. during pregnancy to prevent low birth weight - Intervention at the facility level to distribute nutritional supplements to pregnant women - Intervention to improve the supply chain for maternal supplements so they can be easily reached by rural and urban health posts |
| **1.1.5** | **Food distribution programs** | Food distribution programs targeting women who are pregnant or lactating | **Intervention examples:**   - Provision of beans, grains or other foods as part of a food-based transfer program   **Coding notes:**   - This code should NOT include balanced energy protein supplements, but other foods that are not specialized nutritious foods |
| **1.1.5** | **Family planning/birth spacing** | Provision of family planning services and education on family planning and birth spacing ^1^ | **Intervention examples:**   - Education of health workers, community workers or caregivers on importance of birth spacing to promote optimal maternal and child health - Intervention at the facility level to distribute family planning or to conduct counseling on birth spacing - Intervention to improve the supply chain for family planning so they can be easily reached by rural and urban health posts |
| **1.2.** | **Early newborn** | **Definition of evidence-based recommendation** | **When to use code**  ** Should be used to code program summary* |
| **1.2.1** | **Kangaroo mother care** | Programs that seek to encourage Kangaroo care (i.e., *where the infant is held skin-to-skin with his mother, father, or substitute caregiver*) for babies born at term or low birth weight ^1^ | **Intervention examples:**   - Education interventions for health workers, community, or caregivers that promote kangaroo care especially for premature infants to prevent infant mortality |
| **1.2.2** | **Delayed cord clamping** | Programs that seek to promote delayed cord clamping (i.e., *umbilical cord is not clamped or cut until after it stops pulsating*) during delivery ^1^ | **Intervention examples:**   - Education interventions for health workers |
| **1.2.3.** | **Probiotics** | - Use of probiotics for pre-term infants |  |
| **1.2.4.** | **Emollient** | - Emollient use such as coconut oil or other lubricants for preterm and low birth weight infants. This helps improve skin integrity and prevent infection |  |
| **1.3** | **Child** | **Definition of evidence-based recommendation** | **When to use code**  ** Should be used to code program summary* |
| **1.3.1** | **Early initiation of breastfeeding** | Nutrition education on early (i.e., *child put the breast at least 1 hr. after birth*) ^1^, exclusive (i.e., *no water or food given prior to 6 months of age other than breastmilk*), or continued breastfeeding (i.e., *continued breastfeeding to 1 - 2 years of age*) initiation | **Intervention examples:**   - Education interventions for health workers, caregivers, or community members on the importance of early initiation breastfeeding for infant immunity or birth control - Care groups go to every house with pregnant women age to educate them on the importance of early initiation of breastfeeding and the importance of colostrum |
| **1.3.2** | **Complementary feeding education/promotion *without* provision of foods** | Complementary feeding counseling on appropriate diet for children 6 – 23 months^1^. This education can include the proper types of foods, consistency of foods, number of meals and the appropriate age to introduce foods (i.e., 6 months) | **Intervention examples:**   - Education interventions for health workers, caregivers, or community members - This can also interventions involving food demonstrations, or education provided at growth monitoring visists regarding optimal nutrition - This also involves programs that promote animal source (flesh food, fish, eggs) food consumption among children 6 – 23 mon   **Coding notes:**   - If foods such as small quantity lipid nutrient supplement or CBS+ or CBS++ are provided then this should be coded as 2.3.3. - This intervention should only be coded if the population is **food secure** based on the program participation criteria |
| **1.3.3** | **Complementary feeding education/promotion *with* provision of foods** | Complementary feeding counseling on appropriate diet for children 6 – 23 months^1^. | **Intervention examples:**   - Education interventions for health workers, caregivers, or community members who provide nutrition education and provision of specalized nutritous foods (e.g., small quantity lipid nutrient supplement or CBS+ or CBS++ ) or food transfers (e.g., rice, beans etc.)   **Coding notes:**   - This education can include the proper types of foods, consistency of foods, number of meals and the proper age to introduce foods (i.e., 6 months) accompanied with the provision of foods - If foods such as small quantity lipid nutrient supplement or CBS+ or CBS++ are provided then this code should be used - This intervention should only be coded if the population is **food in-secure** based on the program participation criteria (this is the majority of households in Guinea) |
| **1.3.4** | **Vitamin A supplementation** | Programs to improve Vitamin A supplementation coverage for children 1 – 59 months of age^1^ | **Intervention examples:**   - Education of health workers, community workers or caregivers on importance of vitamin A supplementation for child immunity and eye health - Intervention at the facility level to distribute Vitamin A to children - Intervention to improve the supply chain for Vitamin A supplements so they can be easily reached by rural and urban health posts |
| **1.3.5** | **Zinc supplementation** | Programs that seek to improve Zinc supplementation recommended for children 1 – 59 either after a diarrheal episode ^1^ | **Intervention examples:**   - Education of health workers, community workers or caregivers on importance of zinc supplementation especially during diarrheal episodes - Intervention at the facility level to distribute zinc to pregnant women - Intervention to improve the supply chain for zinc supplements so they can be easily reached by rural and urban health posts |
| **1.3.6** | **Micronutrient powder (MNP)** | Programs that seek to improve micronutrient powered supplementation for 1 – 59-month year old reduces anemia, iron deficiency, anemia, and diarrhea ^1^  **Supplement names include:**   - Multiple Micronutrient Powder - Sprinkles - Micronutrient powder | **Intervention examples:**   - Education of health workers, community workers or caregivers on importance of multiple micronutrient powders for children to prevent anemia - Intervention at the facility level to distribute multiple micronutrients - Intervention to improve the supply chain for multiple micronutrients supplements so they can be easily reached by rural and urban health posts |
| **1.3.7** | **Small or medium quantity lipid nutrient supplements** | Programs that distributed small quantity lipid or medium quantity lipid nutrients to prevent NOT treat malnutrition^1^  **Supplement types include:**   - Small quantity lipid nutrient supplements (SQ-LNS) - Medium quantity lipid nutrient supplements (SQ-LNS)   See other samples here: <https://www.nutriset.fr/en/products> | **Intervention examples:**   - Prevention intervention during lean season involving SQ-LNS to prevent malnutrition |
| **1.3.8** | **Ready to use therapeutic foods (RUTF)** | Treatment of severe acute malnutrition and mild acute malnutrition with RUTF ^1^ | **Intervention examples:**   - Treatment of severe acute malnutrition by distributing RUTF to children < 5 at the facility - Screening at the community level to find children who have severe acute malnutrition to enroll them in treatment - Training of health workers and facility workers on how to treat severe acute malnutrition |
| **1.3.9** | **Insecticide-treated bed nets** | Programs to increase use of insecticide-treated bed nets for the control of malaria among **pregnant women and children under the age of 5** ^1^ | **Intervention examples:**   - Distribution of insecticide treated bed nets to pregnant women and children at the facility level - Education provided to health workers or caregivers on the importance of using a bed net to prevent malaria |
| **1.10** | **Other programing** | Other programing that is delivered through the health system that you think influences child nutrition |  |
| **2.0** | **WASH system** | **Definition of evidence-based recommendation** | **When to use code**  ** Should be used to code program summary* |
| **2.1** | **Water access** | Seeking to increase access to improved water sources ^2^ | **Intervention examples:**   - Establishment/ building of improved water sources within a community   **Coding notes:**   - **Improved water sources include:** Public taps or standpipes, tube wells or boreholes, protected dug wells, protected springs, and rainwater collection - **Unimproved water sources:** Unimproved water sources include unprotected wells, unprotected springs, surface water (e.g. river, dam or lake), vendor-provided water, bottled water (unless water for other uses is available from an improved source) and tanker truck-provided water - This code should only be used for drinking water sources and NOT water being used for agiculture |
| **2.2** | **Water quality** | Programs seeking to improve the quality of water through purification (i.e., water that is appripate to drink) | **Intervention examples:**   - Provision of hygiene kits or bleach to improve the quality of water being consumed - Education on the importance of using water treatments prior to consumption |
| **2.3** | **Hygiene promotion** | Handwashing and the importance of hygiene during cooking and feeding (i.e., washing with soap, keeping animals away from cooking area, closed kitchen, covering food while being stored etc.) ^3^ | **Intervention examples:**   - Distribution of hand washing stations during COVID-19 to all households - Education to caregivers on the importance of hand washing during feeding |
| **2.4** | **Sanitation** | Access and use of improve sanitary facility (i.e., pit latrine), end to open defecation  Improved trash management to ensure that the community environment stays clean | **Intervention examples:**   - Building new latrines in a community   **Coding notes:**  **Improve sanitary facility includes:**   - Flush toilet - Connection to a piped sewer system - Connection to a septic system - Flush / pour-flush to a pit latrine - Ventilated improved pit (VIP) latrine |
| **2.4** | **Other programing** | Other programing that is delivered through the WASH system that you think influences child nutrition |  |
| **3.0** | **Food system** | **Definition of evidence-based recommendation** | **When to use code**  ** Should be used to code program summary* |
| **3.1** | **Biofortification of staple crops** | Programs that promote biofortification inovlves breeding staple crops (i.e., rice, corn, cassava, bean, pearl millet, wheat, sweet potatos etc.) through plant breeding or biotechnology to have a richer micronutrient content including: Vitamin A, iron, and zinc ^4^ | **Intervention examples:**   - A program that introduces a new type of rice in a community that is enriched with iron - Education to farmers on the importance of using/growing biofortified crops |
| **3.2** | **Homestead food production** | Programs that promote establishment of home gardens ^3^ or creation of farming groups to promote community gardening ^3^ or the provision of seeds/fertilizer and farming equiptment to increase garden production | **Intervention examples:**   - Seeds and fertilizer are provided to a women’s group to increase garden production in a community with low food security - A training to farmers on different types of irrigation approaches that can improve garden production - The establishment of farming groups within a community and the provision of seeds and farming equipment to improve food security |
| **3.3** | **Livestock programing** | Programs that seek to improve knowledge of livestock rearing, and processing ^3^  Provision of dairy cows, goats, sheep, pigs, chickens, fish or other small scale livestock to improve household food security ^3^ | **Intervention examples:**   - Intervention provides sheeps, cows or goats to promote livestock rearing - Education on lifestock rearing to improve productivity of breed |
| **3.4** | **Food fortification programing** | Food foritification programing including salt iodization, and fortification of grains, maize, potatoes or other foods with iron, vitamin B12, Folic Acid, Vitamin A and Zinc ^5,6^ | **Intervention examples:**   - All flours are fortified with iron and folic acid to prevent neural tube defects |
| **3.5** | **Improving food value chains** | Programs that seek to improved coordination between different actors in the local food supply chain (e.g., linking school feeding programs to homestead or community food production to improve income of local households) ^7^ | **Intervention examples:**   - This can includes connecting/supporting household or small scale garden or livestock by connecting them to larger markets |
| **3.6** | **Irrigation** | Programs that seek to improve water for farmers | **Intervention examples:**   - Water sheds used to store water and can be used to water homestead crops or gardens   **Coding notes:**   - **Types of irrigation include:**Water tanks used for irrigations, sprinkers, canals, surface irrigation, drip irrigation systems |
| **3.7** | **Market access** | Programs that seek to decrease distance to markets such as building roads to connect markets, installation of new markets | **Intervention examples:**   - New market established in rural town where there is limited access to fresh foods and markets |
| **3.8** | **Other programing** | Other programing that is delivered through the food system that you think influences child nutrition |  |
| **4.0** | **Social protection** | **Definition of evidence-based recommendation** | **When to use code**  ** Should be used to code program summary* |
| **4.1** | **Women’s empowerment activities** | Programs that seek to improve women’s empowerment through income generation/access to resources/or increased time, and increased decision making power and opportunities for skill development or education ^8^ | **Intervention examples:**   - Activities can seek to improve: women’s education, asset ownership, independent decision making   **Coding notes:**   - Do not include reduction in FGM, child marriage or GBV |
| **4.2** | **Cash transfers/resource** | Conditional cash transfers (i.e., *have to received education or participating in programing before receiving money transfer*) and non-conditional cash transfer (i.e., money transfer provided to person or household based on characteristics no activities included) to vulnerable households.^7^ | **Intervention examples:**   - Vulnerable households identified during covid received a cash trasfer to improve their households food security (non-conditional) including pregant and lactating women |
| **4.3** | **Other social protection programing** | Other programing that is delivered through the social system that you think influences child nutrition |  |

**References**

1.	Bhutta ZA, Das JK. Interventions to address maternal and childhood undernutrition: current evidence. *Nestle Nutrition Institute workshop series*. 2014;78:59-69. doi:10.1159/000354941

2.	Heidkamp RA, Piwoz E, Gillespie S, et al. Mobilising evidence, data, and resources to achieve global maternal and child undernutrition targets and the Sustainable Development Goals: an agenda for action. *Lancet*. Apr 10 2021;397(10282):1400-1418. doi:10.1016/s0140-6736(21)00568-7

3.	Improving Young Children’s Diets During the Complementary Feeding Period (UNICEF) (2020).

4.	Ruel MT, Quisumbing AR, Balagamwala M. Nutrition-sensitive agriculture: What have we learned so far? *Global Food Security*. 2018/06/01/ 2018;17:128-153. doi:<https://doi.org/10.1016/j.gfs.2018.01.002>

5.	WHO F, UNICEF, GAIN, MI, & FFI,. *Recommendations on Wheat and Maize Flour Fortification Meeting Report: Interim Consensus Statement*. 2009. Accessed December 16, 2022. <https://www.who.int/publications/i/item/WHO-NMH-NHD-MNM-09.1>

6.	Luthringer CL, Rowe LA, Vossenaar M, Garrett GS. Regulatory Monitoring of Fortified Foods: Identifying Barriers and Good Practices. *Global health, science and practice*. Sep 2 2015;3(3):446-61. doi:10.9745/ghsp-d-15-00171

7.	Ruel MT, Alderman H. Nutrition-sensitive interventions and programmes: how can they help to accelerate progress in improving maternal and child nutrition? *The Lancet*. 2013;382(9891):536-551. doi:10.1016/S0140-6736(13)60843-0

8.	Santoso MV, Kerr RB, Hoddinott J, Garigipati P, Olmos S, Young SL. Role of Women's Empowerment in Child Nutrition Outcomes: A Systematic Review. *Advances in Nutrition*. 2019;10(6):1138-1151. doi:10.1093/advances/nmz056 %J Advances in Nutrition

**Appendix B. Qualitative codebook – Implementation science framework for nutrition**

| **CODE NO.** | **BRIEF CODE NAME** | **DESCRIPTION** | **WHEN TO USE CODE** |
| --- | --- | --- | --- |
| **1.0** | **Factors shaping programs** | Factors shaping the implementation of evidence-based programs found to improve maternal and child nutritional status based on the current literature (See Appendix P for a list of evidence based programs relevant to this codebook) |  |
| **1.1** | **Enabling environment factors** | The enabling environment factors are defined as policy frameworks, governance, finances, private sector organizations, and stakeholder dynamics that influence the implementation of evidence based programs | This code should be used to describe the presence or lack of presence of a policy, availability of government funding, and alignment or misalignment of stakeholders' objectives that ultimately shape evidence based program implementation |
| **1.2** | **Implementing organization factors** | Implementing organization factors are defined as organizational characteristics including leadership, commitment, readiness, priorities, incentives capacity, and accountability. This domain also encompasses staff characteristics of each organization including their knowledge, skills, beliefs, workload, self-efficacy, resources, and accountability that can influence implementation of evidence based programs | This code should be used to describe the ability of organizations and their staff to carry out programs including facilitators or barriers such as knowledge, workload, self-efficacy, resources, and accountability.  If funding influences an organization's ability to meet its needs to carry out programs (e.g., adequate staff, and resources) the text can be double coded with code 1.1.  Advocacy carried out by organizations for new policies not currently in place can also be coded here. |
| **1.3** | **Implementation process factors** | Implementation process factors are defined as the initiation, planning, scale-up, and sustained commitment to carry out a program. | This code should be used to describe barriers or facilitators to the implementation process such as planning before implementation, supply chains, ability to expand the program, and sustainability of programming.  Code 1.1. can also be double-coded here if it is the primary reason why the implementation process is strong or poor is due to funding. |
| **1.4** | **Individual, household, and community factors** | The beneficiary domain or the individuals, households, and community level factors are the needs, resources, capacities, and any behavioral, economic, and political factors that influence the uptake of evidence based programs | This code should be used to describe any beneficiary-related facilitators or barriers to program implementation such as misalignment of program objectives with community norms, and lack of roads to access program activities. |
| **2.0** | **Recommendations for program implementation** | Recommendations that stakeholders have for improving the implementation of evidence-based programs found to improve maternal and child nutrition (See Appendix P for a list of evidence based programs) |  |
| **2.1** | **Enabling environment recommendations** | Recommendations by stakeholders to improve the enabling environment, policy frameworks, governance, and stakeholder dynamics to support the implementation of evidence-based programs to improve maternal and child nutrition. | This code should be used to describe any recommendations that stakeholders have to improve the policy environment such as the introduction of a new policy, strategies to overcome funding gaps, or improved alignment of stakeholders goals and objectives. |
| **2.2** | **Implementing organization recommendations** | Recommendations made by stakeholders to improve organizational characteristics including leadership, commitment, readiness, priorities, incentives capacity, and accountability. This domain also encompasses staff characteristics of each organization including their knowledge, skills, beliefs, workload, self-efficacy, resources, and accountability that may ultimately improve implementation of programs | This code should be used to describe any recommendations that stakeholders have to improve program implementation through current implementing organizations or their staff such as training, accountability strategies, provision of additional resources, or skill transfers. |
| **2.3** | **Implementation process recommendations** | Recommendations to improve program implementation including the initiation, planning, scale-up, and sustained commitment of programs | This code should be used to to describe any stakeholder recommendations for improving the implementation process such as formative research strategies, program planning, evaluation, or sustainability strategies. |

**Appendix C. Salient factors shaping program implementation**

| **Implementation domain** | **Factors described to shape program implementation during interviews** |
| --- | --- |
| **Enabling environment** |  |
| Finances | - Lack of funding to operationalize certain policies (e.g., Multisectoral Nutrition Strategic Plan, universal clean water access, social protections) - Reliance on donors for large scale programming (e.g., Vitamin A Supplementation) |
| Policy framework | - Policy frameworks are strong in some areas (e.g., Multisectoral Nutrition Policy, expanding food quality control) - Policy frameworks are suboptimal in other areas (e.g., social protection strategy poorly developed, water regulations, social protection for farmers, strategy to improve food security during lean season) |
| Dynamics and alignment | - Poor cross sectoral collaboration leads to delivery of vertical (i.e., soiled programming from national to community level) instead of integrated programming due to lack of knowledge of programming in other sectors (e.g., health and social protection system) - Miss alignment exists between funders and implementors (e.g., nutrition not valued, and donors focus on other things) |
| Governance | - Government often uses budgets to support only certain sectors such as agricultural and mining - Socio-political barriers including corruption prevent mobilization of funds for programs across systems - Poor regulation results in overfishing, mining, and deforestation which all contribute to degradation of naturally available resources |
| Global factors | - COVID-19 and Ebola related constraints disrupted health programming and movement related restrictions limited food trade between municipalities |
| **Implementing organization** |  |
| Leadership | - Organizations advocate for development of policies including free healthcare the vulnerable and routine evaluation of political commitments and national programs |
| Commitment | - Lack of ownership of programming at all levels of implementation leads to poor programming - The actors responsible for implementing programs are facilitators especially community relays who work at the decentralized level with little resources or financial incentive |
| Knowledge and skills | - Lack of staff trained on nutrition at the decentralized level - Lack of guidelines and training pertaining to policies at the operational level to facilitate implementation - Suboptimal resources to carryout trainings that require food, accommodation, and per diem - Non-governmental organizations support trainings (e.g., emergency obstetric neonatal care), but working within the educational system (e.g., Midwifery school) to improve training opportunities for the next generation of the workforce can be challenging - Recruitment of civil servants without proper training (e.g., someone with a degree in economics as social worker) to fulfill their roles - Miss alignment in terminology and cross system knowledge (e.g., difference between food security, food, and nutrition) - Trained staff act as a resource to organizations, government, and community members in their area |
| Workload | - Human resources (e.g., civil worker to population ratio) - Especially in the health system, staff are often responsible for several programs (e.g., nutrition and immunization) which is often not feasible for such large programs |
| Management | - Some organizations have clear communication lines for addressing issues (e.g., Société des Eaux de Guinée) - When employees leave, there is a delay in programming and poor follow-up on activities |
| Incentives | - Staff ‘chase’ projects due to make money and because they are under poor management |
| **Implementation process** |  |
| Initiating, scoping and engaging | - Engaging with local development committees, NGOs and authorities facilitates skill transfers, community ownership, and program implementation - Participatory approaches to programming that include community members at the ground level may improve program planning and implementation strategies |
| Planning (e.g., formative research, design) | - Lack of formative research or pilots to better cater programming leads to community conflicts, suboptimal use of program funds, and inability to use evidence to choose intervention platforms and channels - Many policies and strategies include nutrition, but the programs implemented don’t include nutrition components (e.g., social protections, agriculture) - Technical meetings between partners are needed to avoid duplication of activities and to discuss potential bottle necks and solutions based on previous programs (e.g., Vitamin A programming) - Lack of integration of services within systems (e.g., malaria testing and severe acute malnutrition screening) and across systems (e.g., health and social protections) - Intervention designs don’t go beyond awareness which is not sufficient to promote behavior change |
| Implementation, scale-up, improvements | - Suboptimal implementation of planned activities (e.g., sensitization at health facilities, universal healthcare coverage) - Poor uptake of programs within the health sector (e.g., stop attending routine child health clinics after 11 months, severe acute malnutrition treatment) - Poor supply chains and lack of program inputs limit coverage (e.g., ready to use supplemental food, Vitamin A) - Need for scale up of effective interventions (e.g., 1,000 days project, 40 communes of convergence project, Infant and young child feeding (i.e., ANJE)) - Maintenance and expansion of basic resources is needed for growing population (e.g., water taps, latrines) - Strong monitoring and evaluation including impact and process evaluation help to identify efficacious programs and breaks in the system, respectively. - National monitoring systems like DHIS2 help track health data at the national level, but there is not a platform for all systems and none of the current platforms allow for real-time access to monitoring data - Poor process evaluation leads to inability to make data driven decisions and intervention adjustments |
| Commitment, support, financing and sustainability | - Lack of financial means to pay staff and to scale up interventions (e.g., social protections services, ANJE) - Lack of adequate funding to buy necessary program inputs to meet population needs (e.g., essential health services) - Poor intersectoral commitment despite routine meetings and clear plans (e.g., Multisectoral Nutrition Policy) |
| **Individual, household, community** |  |
| Community norms | - Dietary norms such as infant and young child food restrictions (i.e., taboos), lack philosophy to provide a diverse diet, poor food utilization, early introduction of water before 6 months of age, preference for formula milks - Belief among young people that agriculture is not a job |
| Resources | - Lack of access to basic water, sanitation, and health services results in suboptimal behaviors (e.g., drinking rainwater, open defecation, use of traditional medicine) - Household financial constraints limit agricultural activity, healthcare seeking practices, and purchase of enough food to provide a healthy diet for the household - Poor road systems limit food trade between municipalities and healthcare access |
| Behavioral | - Community ownership in programming (e.g., village savings loan group) is a facilitator to program implementation |
